# Astaxanthin-Loaded Nanostructured Lipid Carriers for Preservation of Antioxidant Activity

**DOI:** 10.3390/molecules23102601

**Published:** 2018-10-11

**Authors:** Violeta Rodriguez-Ruiz, José Ángel Salatti-Dorado, Abolfazl Barzegari, Alba Nicolas-Boluda, Amel Houaoui, Carmen Caballo, Noelia Caballero-Casero, Dolores Sicilia, Jorge Bastias Venegas, Emmanuel Pauthe, Yadollah Omidi, Didier Letourneur, Soledad Rubio, Virginie Gueguen, Graciela Pavon-Djavid

**Affiliations:** 1 ERRMECe Laboratory, Biomaterials for Health Group, University of Cergy Pontoise, Maison Internationale de la Recherche, I MAT, 1 rue Descartes, 95031 Neuville sur Oise, France; amel.houaoui@u-cergy.fr (A.H.); emmanuel.pauthe@u-cergy.fr (E.P.); 2 Department of Analytical Chemistry, Institute of Fine Chemistry and Nanochemistry, Campus of Rabanales, University of Cordoba, 14071 Cordoba, Spain; j_a_salatti@hotmail.com (J.Á.S.-D.); calic_amb@hotmail.es (C.C.); a42caasn@uco.es (N.C.-C.); qa1sicrm@uco.es (D.S.); qa1rubrs@uco.es (S.R.); 3 INSERM U1148, Laboratory for Vascular Translational Science, University Paris 13, Sorbonne Paris Cit. 99, Av. Jean-Baptiste Clément, 93430 Villetaneuse, France; barzegari.abolfazl@gmail.com (A.B.); alba.nicolas.boluda@gmail.com (A.N.-B.); didier.letourneur@inserm.fr (D.L.); virginie.gueguen@univ-paris13.fr (V.G.); graciela.pavon@univ-paris13.fr (G.P.-D.); 4 CORDUNAP IBT, Avenida Playa Brava 3256, Iquique 1100000, Chile; jorge.bastias@cordunap.cl; 5 Research Center for Pharmaceutical Nanotechnology, Tabriz University of Medical Sciences, Tabriz 51368, Iran; yadollah.omidi@gmail.com

**Keywords:** astaxanthin, nanostructured lipid carriers, antioxidants, *Haematoccocus pluvialis*, α-TEAC, lipophilic ABTS assay

## Abstract

Astaxanthin is a xanthophyll carotenoid showing efficient scavenging ability and represents an interesting candidate in the development of new therapies for preventing and treating oxidative stress-related pathologies. However, its high lipophilicity and thermolability often limits its antioxidant efficacy in human applications. Here, we developed a formulation of lipid carriers to protect astaxanthin’s antioxidant activity. The synthesis of natural astaxanthin-loaded nanostructured lipid carriers using a green process with sunflower oil as liquid lipid is presented. Their antioxidant activity was measured by α-Tocopherol Equivalent Antioxidant Capacity assay and was compared to those of both natural astaxanthin and α-tocopherol. Characterizations by dynamic light scattering, atomic force microscopy, and scattering electron microscopy techniques were carried out and showed spherical and surface negative charged particles with z-average and polydispersity values of ~60 nm and ~0.3, respectively. Astaxanthin loading was also investigated showing an astaxanthin recovery of more than 90% after synthesis of nanostructured lipid carriers. These results demonstrate the capability of the formulation to stabilize astaxanthin molecule and preserve and enhance the antioxidant activity.

## 1. Introduction

Astaxanthin (3,3′-dihydroxy-β-β’-carotene-4,4′-dione) is a xanthophyll carotenoid mainly produced by microorganisms and marine animals. *Haematococcus pluvialis*, a green microalga, accumulates very high levels of astaxanthin under stressful conditions such as high salinity, nitrogen deficiency, high temperature, and light [1]. Astaxanthin from *Haematococcus pluvialis* presents three forms (free, monoester, and diester) and is commercialized as an oleoresin obtained by extraction of the microalga with supercritical carbon dioxide and solubilized in sunflower oil. The chemical structure of astaxanthin contains two 6C-cycles with a carbonyl and a hydroxy group each separated by an unsaturated chain with eleven carbon–carbon double bonds. This specific configuration confers to astaxanthin as having powerful antioxidant activity on singlet oxygen quenching, thus it is 40 and 100 times more effective as an antioxidant than β-carotene and vitamin E, respectively [2]. The intracellular antioxidant activity of natural extracts of microalgae has proved to be around 90 times higher than synthetic astaxanthin [3]. Several studies have shown a powerful antioxidant and anti-inflammatory activity of natural astaxanthin in vitro on different types of cells [3,4] as well as in animal models [5]. However, until now, all natural antioxidant therapies have failed in clinical trials, likely due to several reasons including the lack of appropriate delivery systems. Indeed, astaxanthin, like other antioxidant molecules, has low solubility in water, high instability, and low bioavailability severely limiting its applications. Moreover, like all carotenoids, astaxanthin is not synthesized by humans, making it necessary for the exogenous supply in the diet [6]. Thus, it is necessary to develop a suitable drug delivery system which has the capability to overcome these limitations.

A promising approach involves nanostructured lipid carriers (NLCs), which offer protection of the active principle ingredient (API) while allowing a controlled release [7,8]. NLC’s consist of a mixture of solid and liquid (oils) lipids which are able to form a solid lipid matrix at physiological temperature. The addition of the liquid lipid into the formulation leads to a low ordered inner structure allowing high loadings of the active ingredients [9]. Moreover, the availability of the API can be improved, due to the new physicochemical parameters of the delivery systems which have shown to be stable in aqueous solution. These lipid carriers can be environmentally-friendly produced without any organic solvent [10]. Easy scale-up of NLC synthesis provides a supplementary argument in favor of cosmetic and nutraceutical NLC applications [11,12,13].

For these reasons, NLC represent an interesting option to preserve antioxidants controlling its release rate. Several studies concerning antioxidant-loaded NLC such as quercetin- [14], lycopene- [15], and curcumin-loaded [16] NLC have been reported.

In literature, few articles are available concerning astaxanthin-loaded NLC [7,8,17]. Most of them focused on the formulation optimization. For example, Tamjidi et al. [7] studied the influence of lipid composition and physical parameters on astaxanthin-loaded NLC. They found that under optimal conditions, degradation of astaxanthin oleoresin was approximately 20% after 25 days of storage [10]. No studies on the antioxidant activity of astaxanthin oleoresin in NLC formulations has been undertaken so far.

Here we report the synthesis of astaxanthin-loaded NLC using sunflower oil as the liquid lipid and the antioxidant activity of the encapsulated astaxanthin oleoresin (natural astaxanthin obtained by supercritical CO_2_ extraction; AstaCO_2_) using the physicochemical lipophilic α-Tocopherol Equivalent Antioxidant Capacity (α-TEAC) assay. Sunflower oil, a usual diluent for commercial astaxanthin oleoresin, was used for this application because it is a very rich source of antioxidants such as vitamin E (440–1520 mg/kg) and phytosterols (2400–5000 mg/kg), which may help to preserve the astaxanthin antioxidant activity. Also, its fatty acid composition profile is quite similar to that of astaxanthin oleoresin. Physicochemical characterization of NLC was carried out by spectrophotometry, dynamic light scattering (DLS), atomic force microscopy (AFM), and scanning electron microscopy (SEM). Evaluation of the antioxidant activity of the astaxanthin-loaded NLC formulations permitted gaining some insights on their chemical stability. Below the most relevant results are presented.

## 2. Results and Discussion

### 2.1. Synthesis and Physicochemical Characterization of NLC

**Figure 1:**
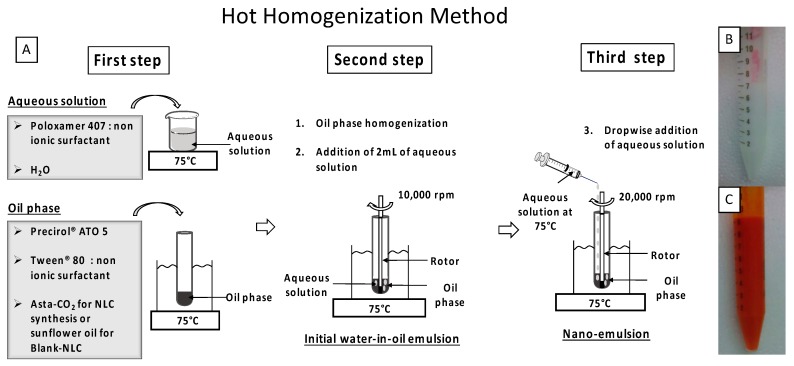
Scheme of synthesis procedure (**A**); macroscopic images of Blank-nanostructured lipid carrier (NLC) (**B**) and AstaCO_2_-NLC (**C**).

Synthesized NLC suspensions are made of a mixture of solid and liquid lipids (Precirol^®^ ATO 5 and sunflower oil, respectively) forming an unstructured solid matrix in an aqueous solution, which contains a mixture of surfactants (Tween^®^ 80 and Poloxamer 407). The NLC components used in the synthesis are all approved by the regulatory agencies [18]. NLC synthesis was carried out by the Hot Homogenization (HH) method (Figure 1A) as described in the Materials and Methods section. After complete addition of aqueous solution over the oil phase, the obtained nanoemulsion was cooled down at room temperature and the pH adjusted (8.4). Homogeneous suspensions of NLC were obtained after 24 h at 4 °C (Figure 1B,C). For NLC containing CO_2_ supercritical extracted natural astaxanthin (AstaCO_2_-NLC) a characteristic orange color was observed (Figure 1C), whereas suspension of astaxanthin-free NLC (Blank-NLC) was white (Figure 1B).

After centrifugation no precipitate or phase separation was observed and all suspensions were opaque suggesting a high percentage of NLC formation (Figure 1B,C). These observations are reinforced by results obtained from quantification of both Blank-NLC and AstaCO_2_-NLC suspensions, which gave similar mass concentrations (average of 79 ± 7 g/L). This value corresponds to 90 ± 11% of initial amount of matter (water excluded), indicating that the major part of all NLC components, including AstaCO_2_, is homogenously distributed in the suspension.

NLC suspensions were characterized by dynamic light scattering (DLS), atomic force microscopy (AFM), and scanning electron microscopy (SEM). The DLS technique gives information about the volumetric mean diameter of particles. The particle size, polydispersity index (PDI), and zeta potential (ZP) were measured by DLS for AstaCO_2_-NLC and Blank-NLC samples (Table 1).

**Figure 2:**
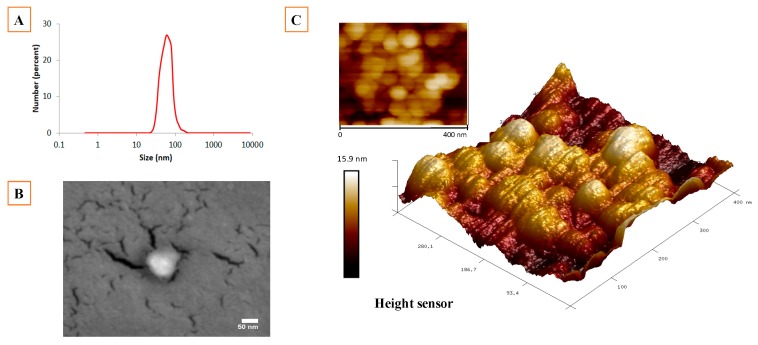
Z-average size (number-based distribution) of AstaCO_2_-NLC measured by DLS (**A**). SEM image of AstaCO_2_-NLC (scale bar 50 nm) (**B**). AFM images of AstaCO_2_-NLC in both topographic mode and 3D mode (**C**).

The average particles size for freshly prepared NLC formulations was 66 nm for Blank-NLC and 60 nm for AstaCO_2_-NLC (Table 1 and Figure 2A). No statistical differences were found between loaded or not loaded NLC. Polydispersity index reflects the difference of primary sizes of nanoparticles in the suspension and their tendency to aggregate [19,20]. PDI values were around 0.3 (Table 1), which is in the same range of those found in the literature [7,21,22]. The synthesis process (HH method) is obviously a critical factor for size distribution of NLC suspension however the nature and content of lipids as well as the molecular weight surfactant employed play an important role in the stability of colloidal system [23,24]. Concerning zeta potential, there are several parameters such as chemical composition, sample concentration, solvent, and pH that can influence the surface charge values. From a physicochemical point of view, large ZP values (above +30 mV and below −30 mV) improve stability in a solution since repulsive forces among particles are stronger when the surface charge is higher. ZP values around 0 mV could indicate instability and flocculation. According to ZP measurements (~25 mV, Table 1), both AstaCO_2_-NLC and Blank-NLC suspensions can be considered stable [25].

Stability studies were performed by storing NLC samples at 4 °C, protected from light, for 1 month. According to Table 1, all NLC samples seem to be stable over a month since their size and PDI were not significantly changed. ZP values of both samples (AstaCO_2_-NLC and Blank-NLC) reminded stable too (Table 1).

Morphology of NLC was studied by liquid atomic force microscopy (AFM) technique. NLC suspensions were studied at hydrated state in order to conserve the original structure. AFM images revealed spherical NLC structures (Figure 2C). The size and shape of the NLCs were also evaluated by scanning electron microscopy (SEM), showing the same spherical tendency and being in the nanosize range (Figure 2B). NLC sizes were in accordance with DLS measurements. It should be noted that SEM sample preparation procedures could modify the NLC shape due to the drying process and vacuum [18,26].

### 2.2. Evaluation of Astaxanthin Content in AstaCO_2_ and AstaCO_2_-NLC

In order to determine astaxanthin content in AstaCO_2_ and AstaCO_2_-NLC samples, natural astaxanthin (AstaN) was used as standard for astaxanthin calibration curve. AstaN spectrum shows a maximum absorption (λ_max_) at 480 nm and an extinction coefficient (ε_480nm_) of 1.35 × 10^5^ L mol^−1^ cm^−1^ in EtOH/DCM (% *v*/*v*; 60/40). This extinction coefficient (ε_480nm_) was used to calculate the astaxanthin content in the AstaCO_2_ sample previously diluted in the same solvent. Spectrophotometric results showed an astaxanthin concentration of 54 mM in AstaCO_2_.

In order to assess the astaxanthin recovery in NLC suspensions, spectrophotometric measurements (see 3.8 Beer–Lambert law parameters and 3.9 Determination of the astaxanthin concentration in AstaCO_2_ and AstaCO_2_-NLC sections of Materials and Methods) were performed showing an astaxanthin recovery of 93 ± 2% after synthesis of NLC suspensions. A stability study showed that the astaxanthin content remains at 90 ± 5% after 1 month of storage. These results confirm higher preservation of astaxanthin in the investigated NLC compared with that obtained in the NLC previously reported [7] (approximately 20% of degradation after 25 days of storage), which could be a result of the presence of antioxidants in the sunflower oil.

### 2.3. Antioxidant Activity of AstaCO_2_-NLC by Lipophilic ABTS Assay

**Figure 3:**
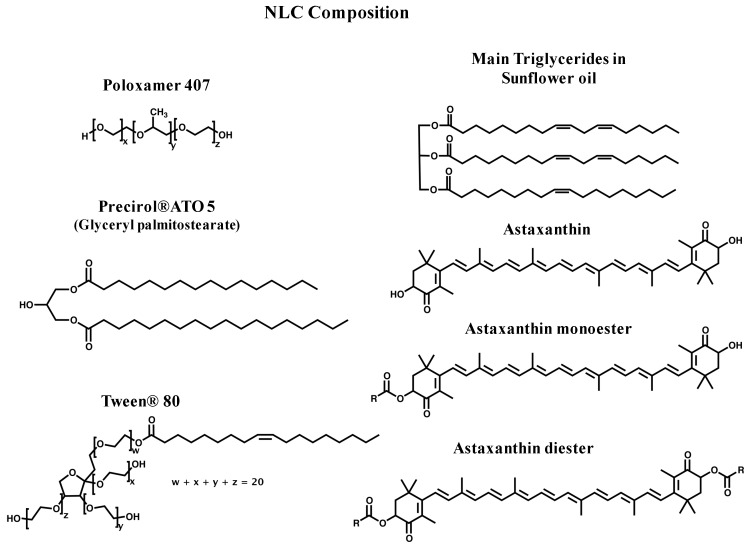
Chemical structures of all components of AstaCO_2_-NLC formulations.

AstaCO_2_ obtained from *Haematococcus pluvialis* consists of lipid soluble fatty acids (~90%) and carotenoids (~10%). The fatty acid fraction mainly contains linoleic (C18:2 cis, ~31%), oleic (C18:1 cis, ~24%), γ-linolenic (C18:3 cis, ~15%), palmitic (C16:0, ~12%), and eicosatrienoic (C20:3 cis, ~8%) acids. The carotenoid fraction contains astaxanthin in three forms (~5% free, ~70% monoesters, and ~10% diesters, see structures in Figure 3) and other carotenoids (e.g., β-carotene, canthaxanthin, and lutein). Carotenoids, according to their structure, can scavenge free radicals by different pathways: electron transfer, yielding carotenoid radical cations, hydrogen atom transfer from cyclohexene rings, giving neutral radicals, and/or radical addition which gives rise to carotenyl adduct radicals [27,28]. The antioxidant activity of carotenoids mainly depends on their structure (e.g., number of double bonds, type and number of functional groups, esterification, etc.), whereas the particular mechanism for scavenging free radicals is basically related to the nature of the free radical and the environment (e.g., solvent polarity). Electrochemical studies involving astaxanthin (free, monoesters, and diesters) suggest a similar scavenging rate of the esters of astaxanthin toward OH^●^, CH3^●^, and OOH^●^ radicals when compared to astaxanthin itself [29].

The NLC formulations reported here were made up of the surfactants poloxamer 407 and tween 80, the solid lipid precirol and sunflower oil as the liquid lipid (see structures in Figure 3). Fatty acid composition in sunflower oil is as follows, linoleic acid (C18:2 cis, 62.4%), oleic acid (C18:1 cis, 28.3%), and saturated fatty acids (9.3%, mainly C16:0). This fatty acid profile is quite similar to that of the AstaCO_2_. Sunflower oil is also an excellent source of antioxidants such as vitamin E (α-tocopherol, 440–1520 mg/kg) and phytosterols (2400–5000 mg/kg, mainly β-sitosterol, δ7-stigmasterol, stigmasterol, and campesterol) [30].

Evaluation of the antioxidant activity of both AstaCO_2_-NLC and Blank-NLC was carried out by a lipophilic α-TEAC assay, using the cation radical of 2,2′-azino-bis(3-ethylbenzothiazoline-6-sulfonic acid) diammonium salt (ABTS^●+^) as a photometric probe and α-tocopherol as the lipophilic calibration standard. The ABTS/TEAC assay is considered as a mixed electron transfer (ET)-based and hydrogen atom transfer (HAT)-based assay and is particularly suitable for the evaluation of the antioxidant activity of lipophilic compounds such as carotenoids. Another common ET/HAT assay (2,2-di(4-tert-octylphenyl)-1-picrylhydrazyl; DPPH) could not be used for the evaluation of the antioxidant activity of astaxanthin because of the overlapping of the spectral absorbance of this carotenoid with the monitoring wavelength of the radical DPPH^●^ (540 nm) [31].

**Figure 4:**
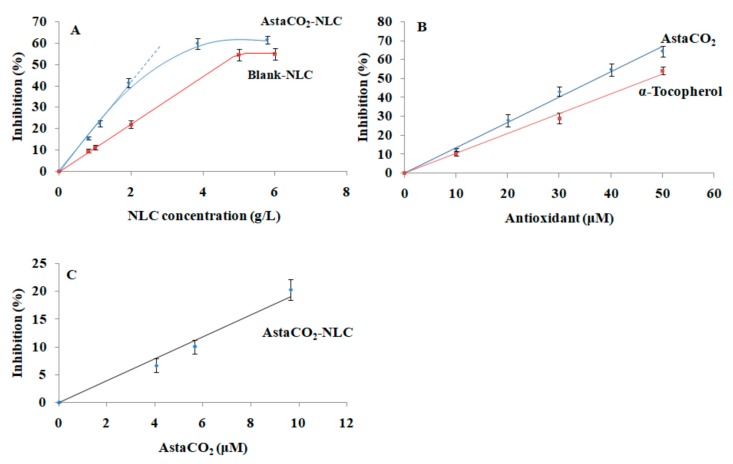
Decrease of the absorbance of 2.2′-azino-bis(3-ethylbenzothiazoline-6-sulphonic acid) diammonium salt radical cation (ABTS^•+^), measured as inhibition, as a function of the concentration of AstaCO_2_-NLC and Blank-NLC, expressed as NLC concentration in (g/L) (**A**); AstaCO_2_ and α-tocopherol, expressed as antioxidant concentration in (µM) (**B**); and AstaCO_2_-NLC, expressed as astaxanthin concentration in (µM) (**C**).

Figure 4A shows the inhibition curves obtained for Blank-NLC and AstaCO_2_-NLC formulations, expressed as concentration in NLC (g/L). They both reached a maximum inhibition from approximately 4 and 5 g/L of NLC for AstaCO_2_-NLC and Blank-NLC, respectively. These values were kept practically constant up to 8 and 15 g/L of NLC (the maximum concentrations tested) for AstaCO_2_-NLC and Blank-NLC, respectively. The slopes of the linear portion of the curves were y = (10.96 ± 0.53)x, R^2^ = 0.998 and y = (20.83 ± 1.04)x, R^2^ = 0.994, for Blank-NLC and AstaCO_2_-NLC, respectively. The slope of the inhibition curve obtained for AstaCO_2_-NLC was almost twice that of Blank-NLC, which indicated the high potential of astaxanthin for radical scavenging. Concerning the Blank-NLC inhibition curve, although both α-tocopherol and phytosterols were considered to be present in these formulations and undoubtedly contributed to the scavenging of ABTS^●+^ cation radicals, their estimated maximum concentration in sunflower oil (around 0.46 µM and 1.6 µM, respectively) was considered too low to account for the total inhibition obtained. The presence of unsaturated lipids in the sunflower oil (e.g., linoleic and oleic acids) which are easily oxidized by different radicals, were considered responsible for the inhibition curve obtained. The easy oxidation of unsaturated lipids has been identified as one of the reasons for increasing the likelihood that carotenoids degrade owing to the attack of radicals produced in lipid oxidation reactions [32]. So, in the light of these results, it seems that formulating NLCs with saturated lipid carriers should give more suitable environments than the unsaturated ones for stabilizing carotenoids. Preliminary experiments carried out by authors involving the encapsulation of astaxanthin in saturated lipid-based NLCs confirm this hypothesis.

Figure 4B shows the inhibition curves for AstaCO_2_ and the calibration standard α-tocopherol, expressed as concentration of antioxidant (µM). Both of them showed a linear behavior in all range of studied concentrations (0–50 µM). The slopes of these graphs were y = (1.02 ± 0.06)x, R^2^ = 0.992, for α-tocopherol and y = (1.35 ± 0.06)x, R^2^ = 0.990 for AstaCO_2_. On the other hand, the slope of the inhibition curve for AstaCO_2_-NLC, expressed as concentration of AstaCO_2_ (µM), and calculated as the difference in the linear portion of the curves for AstaCO_2_-NLC and Blank-NLC (Figure 4C) had a value of y = (1.98 ± 0.25)x, R^2^ = 0.980.

The α-TEAC coefficient (unitless) for both AstaCO_2_ and AstaCO_2_-NLC was calculated as the slope ratio of the respective sample curves vs. α-tocopherol curves. The results shown have a higher antioxidant activity for AstaCO_2_-NLC (α-TEAC = 1.94 ± 0.34) compared to that of AstaCO_2_ (α-TEAC = 1.33 ± 0.14). These results show that stabilization of astaxanthin in NLC formulations confers important scavenging properties and higher antioxidant capacity to astaxanthin. Indeed, the involvement of chemical environment in modulation of antioxidant activity of carotenoids has been previously reported [27,29,33]. In this regard, the capacity of NLC to amplify antioxidant activity was also shown by Nayyer Karimi et al. [16] using DPPH test to evaluate turmeric-NLC scavenging activity compared to free turmeric extract. Furthermore, these results are supported by those obtained in our previous work showing the enhancing of astaxanthin antioxidant capacity by hydroxypropyl-beta-cyclodextrin complexation [34].

## 3. Materials and Methods

### 3.1. Algae Material

Natural astaxanthin from the microalgae *Haematococcus pluvialis* was produced by Pigmentos Naturales (SA, Pica, Tarapaca, Chile). After supercritical CO_2_ extraction (NATECO_2_, Wolnzach, Germany) the extract containing astaxanthin was solubilized in sunflower oil (7%, *w*/*w*, AstaCO_2_) and stored at −20 °C. Natural astaxanthin (AstaN) purchased from Sigma-Aldrich (SML0982; St. Louis, MO, USA) was used as standard.

### 3.2. Chemicals

The following compounds were used in the synthesis of the nanostructured lipid carriers (NLC). Poloxamer 407 was purchased from Sigma-Aldrich (St. Louis, MO, USA); glyceryl palmitostearate (Precirol^®^ ATO 5) was kindly donated by Gattefossé (Nanterre, France); and polyoxyethylene sorbitan monooleate (Tween^®^ 80) was purchased from Carl Roth GMbH (Karlsruhe, Germany). All NLC solutions were formulated using purified water. The following compounds were used in the α-Tocopherol Equivalent Antioxidant Capacity (α-TEAC) assay, (±)-α-tocopherol (≥96%, HPLC), 2,2′-azino-bis(3-ethylbenzothiazoline-6-sulfonic acid) diammonium salt (ABTS), ethanol, and potassium persulfate; all were purchased from Sigma-Aldrich (St. Louis, MO, USA).

### 3.3. Synthesis of AstaCO_2_-Loaded NLC (AstaCO_2_-NLC) by Hot Homogenization (HH) Method

Synthesis of AstaCO_2_-NLC and the appropriate Blank-NLC (astaxanthin-free NLC) were prepared by HH method on the basis of a previously reported methods [7,35]. Optimization of the work-up procedure was performed where it was needed. Specifically, AstaCO_2_ (103 mg) was dissolved at 75 °C in the oil phase, which consists of the solid lipid (Precirol^®^ ATO 5, 450 mg) and Tween^®^ 80 as a surfactant (60 mg). The Blank-NLC was prepared by adding sunflower oil (103 mg). In a separate container, surfactant (Poloxamer 407, 450 mg) was dissolved in purified water (16 mL) (aqueous solution) and heated at 75 °C. The oil phase was homogenized at 10,000 rpm for 2 min at 75 °C (Polytron^®^ system, PT3100 homogenizer with a dispersing aggregate of 7 mm of diameter coupled (Kinematica AG, Luzern, Switzerland). Then, 2 mL of the aqueous solution were added to the oil phase to form the initial water-in-oil emulsion. The rest of the aqueous solution was added dropwise during 10 min into the oil phase under homogenization at 20,000 rpm at constant temperature of 75 °C. After complete addition, the nanoemulsion was cooled down to room temperature (25 °C) for 10 min and the pH was adjusted to pH 8.4. Then the nanoemulsion was stored overnight at 4 °C follow by centrifugation (10,000 rpm, 15 min) to obtain a NLC suspension. A scheme of the synthesis procedure is shown in Figure 1.

### 3.4. Particle Size Analysis and Zeta Potential

Z-average size (number-based distribution), polydispersity index (PDI), and zeta potential (ZP) of NLC were evaluated by using a DLS particle analyser Zetasizer NanoZS (Malvern Instruments Ltd., Worcestershire, UK). For Z-average size measurements, samples were diluted (1:500) with purified water and placed into cuvettes semi micro (Brand Gmbh + Co. Kg, Wertheim, Germany). Light scattering was monitored with an attenuation of 7 at a temperature of 25 °C. For ZP measurements, samples were diluted (1:200) with KCl (1 mM) and placed into a capillary cell (DTS1060). Moreover, these parameters were measured again after 30 days on samples kept at 4 °C. All measurements were performed in triplicate and the data are presented as a mean ± standard deviation (SD).

### 3.5. Atomic Force Microscopy (AFM)

In order to support NLC onto the coverslip, samples were subjected to a soft spin-coating drying (p6700 series, Specialty Coating Systems, Sitek Process Solutions, Rocklin, CA, USA) before addition of ultrapure water droplet for liquid AFM studies. The atomic force microscopy (AFM) experiments were performed in ScanAsyst mode with a Dimension ICON microscope from Bruker (Billerica, MA, USA). All measurements were carried out in ultrapure water at room temperature with a tip model ScanAsyst-Fluid (k = 0.7 N/m, Bruker). Images were analyzed with NanoScope Analysis 1.5.

### 3.6. Scanning Electron Microscopy (SEM)

A thin film of NLC was uniformly deposed on a coverslip by spin-coating. Coverslips were mounted on SEM stubs with carbon tape and gold coated with a sputter coater from Agar Scientific (Stansted, UK) 30 s at 30 mA. NLC samples were then analyzed using a GeminiSEM300 from Carl Zeiss (Jena, Germany) at high-vacuum mode operated at an acceleration voltage of 15 keV using back scattered electrons imaging. Micrographs were finally processed on ImageJ-FIJI software for brightness and contrast adjustment.

### 3.7. Quantification of NLC

Mass concentrations of NLC suspensions were determined by using the following freeze-drying technique. NLC suspensions (500 µL) were introduced in previously weighed vials and frozen at −80 °C overnight. Samples were freeze-dried for 24 h in a Modulyo^®^ Freeze-dryer, F.D. Edwards (Crawley, UK). Then, vials containing NLC freeze-dried materials were weighed again and the amount of dehydrated matter was calculated by weight difference. All experiments were carried out in triplicate.

### 3.8. Beer–Lambert Law Parameters

Spectrophotometric measurements were set up on a Lambda 12 Spectrophotometer (PerkinElmer Inc., Norwalk, CT, USA) in the UV-visible domain (200–800 nm), using a scan speed of 50 nm/min. A calibration curve based on λ_max_ absorption was obtained using AstaN (0–50 μM) in EtOH/DCM (% *v*/*v*; 60/40). The extinction coefficient (ε) was used to calculate the astaxanthin content in AstaCO_2_ and AstaCO_2_-NLC.

### 3.9. Determination of the Astaxanthin Concentration in AstaCO_2_ and AstaCO_2_-NLC

AstaCO_2_ and AstaCO_2_-NLC were diluted and dissolved (% *v*/*v*; 1/5), respectively, in EtOH/DCM (% *v*/*v*; 60/40). AstaCO_2_-NLC samples were centrifuged (10,000 rpm, 5 min) after dissolution to verify the total solubility of NLC. The spectra baseline was monitored on EtOH/DCM (% *v*/*v*; 60/40) for AstaCO_2_ and on Blank-NLC for AstaCO_2_-NLC. Astaxanthin concentrations were determined by UV-visible measurements. All experiments were carried out in triplicate.

For stability studies, astaxanthin recovery was calculated as presented in the following formula.

(1) $$\%\;\mathrm{recovery}=\frac{\left[Astaxanthin\right]\mathrm{final}}{\left[Astaxanthin\right]\mathrm{initial}}\times 100$$

where [Astaxanthin]final corresponds to the concentration of astaxanthin measured in the AstaCO_2_-NLC suspension after dissolution in EtOH/DCM. [Astaxanthin]initial corresponds to the concentration of astaxanthin calculated from the initial amount of AstaCO_2_ added during the synthesis of AstaCO_2_-NLC.

### 3.10. Evaluation of Antioxidant Activity of AstaCO_2_-NLC by α-TEAC Assay

The antioxidant capacities of AstaCO_2_ and AstaCO_2_-NLC were evaluated according to the lipophilic α-Tocopherol Equivalent Antioxidant Capacity (α-TEAC) assay [36,37]. Optimization of the work-up procedure was performed where it was needed. Briefly, a stock solution of the free radical ABTS^•+^ (7 mM) was prepared by mixing (% *v*/*v*; 1/1) ABTS solution (14 mM) and potassium persulfate (K_2_S_2_O_8_, 270.322 g/mol, colorless) solution (4.9 mM) in purified water. The mixture was placed in the dark, at room temperature for 24 h before use. Then, the free radical ABTS^•+^ solution (7 mM) was diluted in purified water in order to reach an absorbance of 0.8 at 733 nm. α-tocopherol was used as the standard antioxidant.

Stock solutions of AstaCO_2_ (50 µM) and α-tocopherol (2 mM) were prepared in EtOH/DCM (% *v*/*v*; 60/40). A range of concentrations was freshly prepared for α-tocopherol and AstaCO_2_ (10–50 µM). AstaCO_2_-NLC and Blank-NLC suspensions in PBS were prepared in the range of 1 to 15 g/L (of lipid content), corresponding to 5 to 75 µM of astaxanthin content in the AstaCO_2_-NLC.

Then, 300 µL of these solutions were added to 1 mL ABTS^•+^ (absorbance 0.8) and incubated 45 min at room temperature under stirring and light protection. All samples were centrifuged (10,000 rpm, 2 min). Then, absorbance was measured ((Abs)_final_, 400–800 nm). Experiments were carried out in triplicate. The inhibition percentage was calculated as presented in the following formula.

(2) $$\%\;\mathrm{inhibition}=\frac{\left({\mathrm{Abs}}_{733\mathrm{nm}}\right)\mathrm{initial}-\left({\mathrm{Abs}}_{733\mathrm{nm}}\right)\mathrm{final}}{\left({\mathrm{Abs}}_{733\mathrm{nm}}\right)\mathrm{initial}}\times 100$$

where $\left({\mathrm{Abs}}_{733\mathrm{nm}}\right)\mathrm{initial}$ corresponds to the Absorbance at 733 nm of the solution obtained by mixing 300 µL of EtOH/DCM (% *v*/*v*; 60/40) with 1 mL of ABTS^•+^ (absorbance 0.8).

To calculate α-TEAC, percentage of inhibition–concentration curves of α-tocopherol and samples were plotted. Then, the α-TEAC coefficient (unitless) is defined as the slope ratio of samples curves to *α*-tocopherol curve.

### 3.11. Statistical Analysis

The mean was used as the measurement of the main tendency, whilst standard deviation (SD) was used to measure dispersion. Results are presented as mean ± SD. A *p*-value < 0.05 was considered significant. Data was analyzed for statistical significance using one-way analysis of variance (ANOVA), followed by Tukey’s HSD post hoc test using JMP software (Version 9; SAS Institute, Cary, NC, USA).

## 4. Conclusions

Previous studies have shown the high antioxidant properties of natural astaxanthin [27,29,33]. This carotenoid has the ability to interact strongly with free radicals as a chain-breaking molecule. However, several disadvantages attributed to its sensitivity to degradation and its low solubilization in physiological systems limit astaxanthin applications. In the present work, we formulated astaxanthin-loaded lipid carriers by using a green chemistry process and adding sunflower oil as the liquid lipid. This study demonstrated the capacity of the nanostructured lipid carrier formulations to stabilize and preserve the astaxanthin antioxidant capacity. Our results strengthen the hypothesis that the lipid environment contributes to the enhancement of the antioxidant capacity of astaxanthin. Indeed, the capacity of NLC to amplify antioxidant activity has already been shown [16].

Green methodology used to prepare lipid-based formulations developed in this work allows more than 90% the astaxanthin recovery in NLC suspensions. AstaCO_2_-NLC suspensions were successfully formulated. Physicochemical characterization by dynamic light scattering showed stable negative charged NLC with z-average and polydispersity values of ~60 nm and ~0.3, respectively, and with spherical morphology confirmed by AFM and SEM.

Owing to these results, AstaCO_2_-NLCs appear to be excellent candidates as antioxidant delivery systems for cosmetics and nutraceuticals as well as for developing new platforms for medical devices applications. Complementary studies are currently being undertaken to develop a greater understanding of the relation between the structures of the astaxanthin loaded NLC and their biointeractions.

## Figures and Tables

**Table 1:** Physicochemical NLC parameters: average particle size (number-based distribution Z-average), polydispersity index (PDI), and zeta potential (ZP) after synthesis (Day 1) and after 1 month (Day 30).

Sample	Z-Average (nm) ^1^		PDI ^1^		ZP (mV) ^1^	
	Day 1	Day 30	Day 1	Day 30	Day 1	Day 30
Blank-NLC	66 ± 17	61 ± 15	0.30 ± 0.03	0.24 ± 0.01	−23.3 ± 1.0	−25.9 ± 0.4
AstaCO_2_-NLC	60 ± 7	57 ± 10	0.33 ± 0.09	0.37 ± 0.05	−25.5 ± 0.7	−23.7 ± 0.4

^1^ n = 3.
